# Supplementary material for: Effectiveness and Safety of Ayurvedic Medicines in Type 2 Diabetes Mellitus Management: A Systematic Review and Meta-Analysis
Source: Front Pharmacol. 2022 Jun 8;13:821810. doi: 10.3389/fphar.2022.821810 (PMC9213670; doi:10.3389/fphar.2022.821810)
Supplement: Supplementary file 1 [file DataSheet1.zip › Supplementary Material/Forest and Funnel Plots/Nigella sativa/TC.pdf]

Nigella sativa - TC

Study	Intervention N	Intervention Mean	Intervention SD	Comparator N	Comparator Mean	Comparator SD	Mean Diff. with 95% CI	Weight (%)
Hosseini 2013	35	242.10	35.70	35	253.90	29.30	-11.80 [-27.10, 3.50]	24.58
Hadi 2015	23	173.00	38.30	20	178.00	43.50	-5.00 [-29.44, 19.44]	16.78
Heshmati 2015	36	195.60	49.12	36	236.10	53.00	-40.50 [-64.11, -16.89]	17.39
Kooshki 2020	27	170.20	47.00	23	203.90	53.80	-33.70 [-61.64, -5.76]	14.47
Jangjo-Borazjani 2021	20	152.90	22.38	20	157.30	19.58	-4.40 [-17.43, 8.63]	26.78
**Overall**							-16.84 [-30.70, -2.97]	

Heterogeneity: $\tau^2 = 142.69$, $I^2 = 59.78\%$, $H^2 = 2.49$

Test of $\theta_i = \theta_j$: Q(4) = 9.42, p = 0.05

Test of $\theta = 0$: z = -2.38, p = 0.02

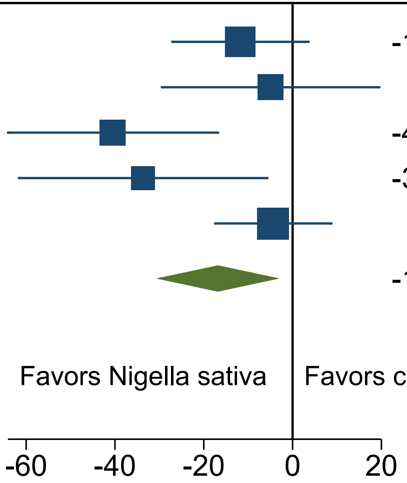

Random-effects REML model
